# Chemotherapy-induced posterior reversible encephalopathy syndrome: Three case reports

**DOI:** 10.1097/MD.0000000000015691

**Published:** 2019-05-13

**Authors:** Bernardo Cacho-Díaz, Nydia A. Lorenzana-Mendoza, Karen Salmerón-Moreno, Gervith Reyes-Soto, Carlos Castillo-Rangel, Roberto Corona-Cedillo, Salvador Escobar-Ceballos, Jaime G. de la Garza-Salazar

**Affiliations:** a Neuro-oncology Unit, Instituto Nacional de Cancerología; b Neurosurgery ISSSTE Hospital Regional 1° de Octubre; c Imaging Department; d Internal Medicine Fundación Clínica Médica Sur; e Research Unit, Instituto Nacional de Cancerología, Mexico City, Mexico.

**Keywords:** cancer, chemotherapy, neuro-oncology, posterior reversible encephalopathy syndrome

## Abstract

**Rationale:**

Posterior reversible encephalopathy syndrome (PRES) has been associated with the use of several medications, including chemotherapeutic agents.

**Patient concerns:**

A 65-year-old woman was diagnosed with adenocarcinoma of the ovary, after sixth-line treatment with topotecan, at the beginning of the fourth cycle, she was admitted to the emergency room for presenting tonic-clonic seizures, visual disturbance, and hypertension. A 66-year-old woman was diagnosed with bilateral breast cancer; due to disease progression, treatment with paclitaxel and gemcitabine was started, 1 month after the last dose of chemotherapy, she was admitted to the emergency room for suffering severe headache, altered mental status, tonic-clonic seizures, and hypertension. A 60-year-old patient diagnosed with breast cancer on the left side, underwent second-line chemotherapy with gemcitabine, carboplatin, and bevacizumab, and 1 month after the last dose of chemotherapy, she was also admitted to the emergency room due to altered mental status, vomiting, tonic-clonic seizures, and hypertension.

**Diagnosis:**

They were diagnosed as PRES based on physical examination, laboratory findings, and imaging techniques that revealed diffuse lesions and edema within the parieto-occipital regions.

**Interventions:**

They received support treatment with blood pressure (BP) control, seizures were controlled with a single anti-epileptic agent, and chemotherapeutic agents from the onset of PRES to its resolution were discontinued.

**Outcomes:**

All these patients improved after medical treatment was started.

**Lessons:**

Medical personnel and therapeutic establishments need to be made aware about this chemotherapy-induced neurologic complication.

## 1 Introduction

Posterior reversible encephalopathy syndrome (PRES) was first described in 1996 by Hinchey et al based on observations in 15 patients with a consistent and reversible pattern of neurological symptoms and neuroimaging findings.^[1]^ PRES represents a clinical radiographic disorder characterized by the presence of subcortical vasogenic edema and white matter predominance lesions in most cases involving the parieto-occipital regions; these symptoms are commonly reversible.^[2,3]^ Although there are no guidelines to direct the assessment, literature indicates that clinical judgement is essential.^[2]^ Diagnosis might be considered in the presence of: transient neurological symptoms with a clinical context of hypertensive crisis, autoimmune disorders, pregnancy-related complications, renal failure, and the use of cytotoxic drugs; along with fully reversible brain edema.^[2,4]^ Clinical manifestations and signs are not specific, however, leading symptoms described are: headache, seizures, altered mental status, and visual impairment; that are usually accompanied by the presence of hypertension.^[2,5]^

We present 3 cases of chemotherapy-associated PRES.

### 1.1 Case 1

**Figure 1:**
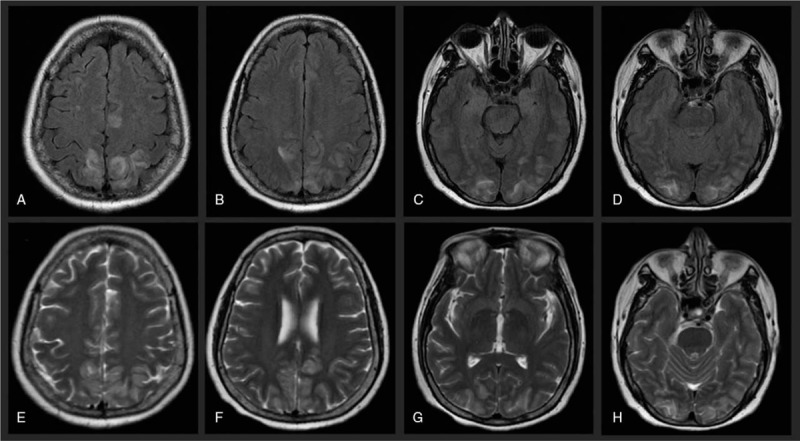
Case 1, MRI showed parieto-occipital hyperintensities on FLAIR (A–D) and T2-WI (E–H).

**Figure 2:**
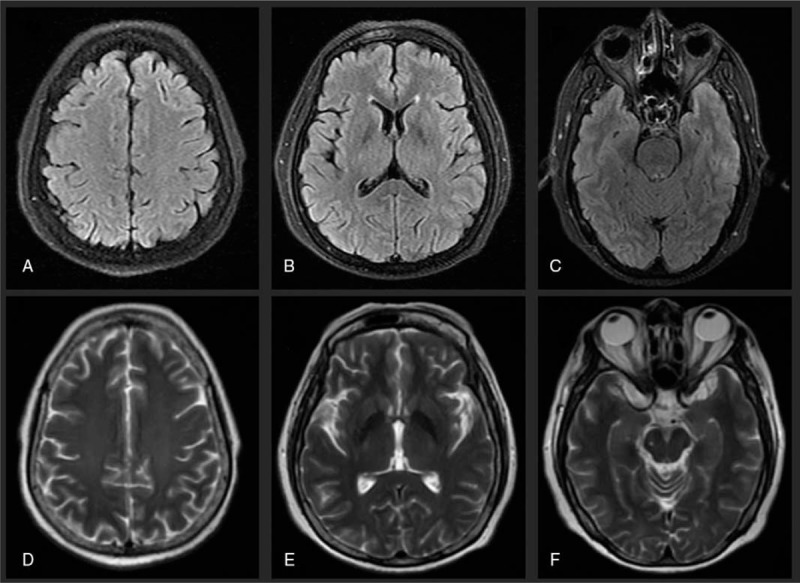
Case 1, 10 days later after treatment initiation, MRI showed resolution of parieto-occipital lesions on FLAIR (A–C) and T2-WI (D–F).

A 65-year-old woman was diagnosed with disseminated endometrioid adenocarcinoma of the ovary. Her past medical history was unremarkable. She was initially treated with right hemicolectomy and oophorectomy followed by 3 cycles of paclitaxel and carboplatin (Taxol/Carbo). Three months later, she underwent interval laparotomy and treatment with 3 more cycles of Taxol/Carbo. After a disease-free period of 23 months, progression localized to the pancreas led to the administration of 3 cycles of Taxol/Carbo. One month later, she relapsed with portal vein and celiac trunk metastatic lesions and was shifted to third-line chemotherapy with 8 cycles of liposomal Adriamycin and underwent cytoreductive laparotomy, achieving a disease-free period of 11 months. Then, she received fourth-line chemotherapy with methotrexate because of disease progression to the liver. She received fifth-line chemotherapy with gemcitabine. Positron emission tomography–computed tomography (PET-CT) demonstrated disease progression, leading to the administration of sixth-line treatment with topotecan (4 mg, total dose). Two days after beginning the fourth cycle, the patient was admitted to the emergency room because of tonic–clonic seizures and visual disturbance. Her blood pressure (BP) was 162/73 mm Hg, and blood tests showed no abnormal findings other than hyperglycemia (174 mg/dL). Physical examination revealed no abnormal findings. She had no medical history of hypertension or diabetes. Brain magnetic resonance imaging (MRI) revealed parieto-occipital hyperintensities on T2-WI and fluid-attenuated inversion recovery as well as restricted diffusion (Fig. 1). Seizures were treated with diazepam and phenytoin (690 mg/d) and strict metabolic and BP control. She was discharged on the 2nd day because of clinical resolution of her symptoms. A new brain MRI was taken 9 days later showing disappearance of the lesions (Fig. 2) leading to the diagnosis of PRES. Although no antihypertensive was prescribed, she did receive levetiracetam (1 g/12 h) for the next 16 months without any report of new seizures; also, she underwent another 3 cycles of topotecan without recurrence of PRES, and finally, eighth-line chemotherapy with Taxol/Carbo was administered with partial response. After 24 months of the diagnosis, she continues treatment under vigilant monitoring by the neuro-oncology unit.

### 1.2 Case 2

**Figure 3:**
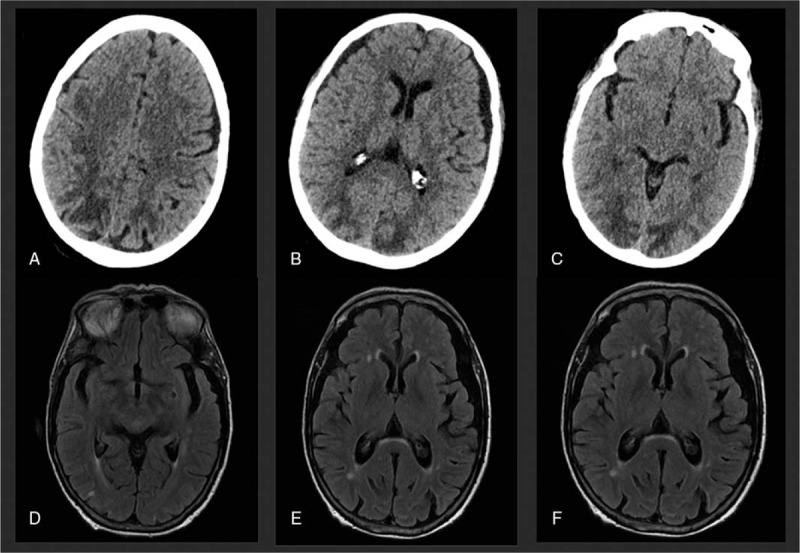
Case 2, CT showed hypodensities in the subcortical parieto-occipital white matter (A–C). Two weeks after treatment initiation, MRI FLAIR showed resolution of the lesions (D–F).

A 66-year-old woman was diagnosed with bilateral breast cancer classified as infiltrating ductal carcinoma hormone sensitive (Luminal A). Her past medical history was unremarkable. She received first-line treatment with neoadjuvant fluorouracil, doxorubicin (Adriamycin), and cyclophosphamide (FAC) prior to surgery; she was, subsequently, consolidated with 4 cycles of vinorelbine (NVB) plus Adriamycin, followed by adjuvant radiotherapy and tamoxifen. One month later, she was diagnosed with uterine cervix carcinoma 1B1, for which she underwent radical hysterectomy. Two years later, she presented with a new mass in the right breast. Core needle biopsy showed infiltrating ductal carcinoma that was treated with neoadjuvant docetaxel plus carboplatin prior to surgery and radiotherapy, and at that time, she received anastrozole treatment. Three years later, her treatment was changed to capecitabine and exemestane because of liver metastases. Four months later, new liver metastases were documented, and NVB was added to capecitabine for another 4 months. Her treatment was changed to docetaxel plus capecitabine leading to partial response. As the patient showed progressive disease, treatment with paclitaxel (120 mg, total dose) and gemcitabine (1200 mg, total dose) was started. One month after the last dose of chemotherapy, she was admitted to the emergency room because of severe headache, altered mental status, tonic–clonic seizures (2 episodes), and hypertension (150/90 mm Hg). Physical examination revealed no abnormal findings. Blood tests showed a lactate dehydrogenase level (LDH) of 819, serum creatinine level of 2.47, and hyperglycemia (184 mg/dL). CT revealed diffuse hypodensities and edema within the parieto-occipital regions (Fig. 3). During hospitalization, she was treated with 840 mg of phenytoin, IV steroids, hydration, and the antihypertensive drug nicardipine (20 mg/12 h). Two days after the event, she remained asymptomatic and was discharged on the 6th day with prescription for long-active nifedipine (20 mg/12 h) and phenytoin (100 mg/8 h); 2 weeks later, MRI and blood tests showed resolution of the abnormalities (Fig. 3), leading to the diagnosis of PRES. Because of development of rash, phenytoin was stopped after 1 month. The patient died after 3 months of diagnosis due to cancer progression (outside the CNS).

### 1.3 Case 3

A 60-year-old patient diagnosed with breast cancer on the left side, classified as infiltrating ductal carcinoma triple negative, was treated with 4 cycles of FAC Ch. Then, she received placlitaxel prior to radical mastectomy. Her past medical history was unremarkable. Eight months later, she presented with headache and nominal aphasia leading to the diagnosis of brain metastasis. The patient underwent radiosurgery and second-line chemotherapy with gemcitabine (1000 mg, total dose), carboplatin (350 mg, total dose), and bevacizumab (1320 mg, total dose). She required steroid to ameliorate the gemcitabine-induced rash, after the last dose of chemotherapy, she presented with altered mental status, vomiting (10 episodes), and tonic–clonic seizure (1 episode) and was admitted to the emergency room.

**Figure 4:**
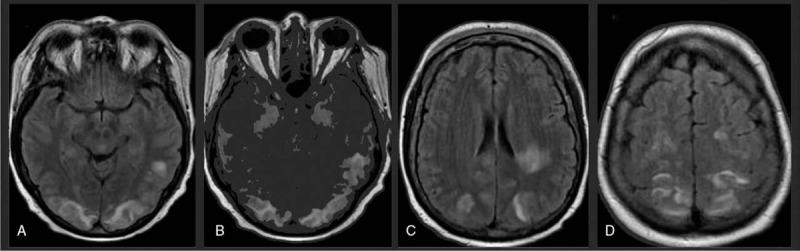
Case 3, MRI showed parieto-occipital hyperintensities on FLAIR (A–D). These were completely resolved a few days after treatment.

Her BP was 150/100 mm Hg. Blood test showed metabolic acidosis, lactate levels of 4.8 mmol/L, platelet count of 106,000 per mL, and hyperglycemia (198 mg/dL). Physical examination revealed no abnormal findings. She was treated with 5 mg of diazepam, IV steroid, hydration, diuretic (20 mg/24 h of furosemide), and antihypertensive drugs. MRI revealed diffuse hyperintensities and edema within the parieto-occipital regions, leading to the diagnosis of PRES probably secondary to bevacizumab treatment (Fig. 4). Diazepam was changed to levetiracetam (1 g/12 h) and after the 2nd day of the event, she showed complete resolution of symptoms and was discharged. Because the patient had completed the last chemotherapy regimen, she received third-line chemotherapy with ixabepilone and underwent radiosurgery to treat brain metastasis progression. The patient died 1 year after the diagnosis of PRES because of complications caused by liver, brain, and soft tissue metastases.

## 2 Discussion

PRES has been increasingly recognized within the cancer population. Chemotherapeutic agents that have previously been described to be associated with PRES include taxanes, platinum derivatives, vinca alkaloids, antimetabolites, anthracyclines, angiogenic inhibitors, folate antagonists, and immunosuppressants.^[6–8]^ Leading theory of the pathophysiological changes in PRES states that rapidly developing hypertension exceeds the upper limit of cerebral blood flow autoregulation and causes hyperperfusion, breaking the blood–brain barrier and allowing the interstitial extravasation of plasma and macromolecules.^[2]^ On the grounds that patients with cancer are exposed to several treatment regimens that include a combination of various cytotoxic agents, it is difficult to identify a direct association between PRES and any of the chemotherapeutic agents in these cases; nonetheless, it has been noted that an intense treatment can increase the risk of PRES as well as the mortality rates.^[9]^

On hospital admission, our patients presented with common symptoms of PRES, including high BP, altered mental status, and seizures. Their neuroimaging findings involving both the hemispheres at parieto-occipital regions were documented as well. Chemotherapeutic agents from the onset of PRES to its resolution were discontinued. All patients showed complete clinical and radiologic resolution of PRES. Despite the risk of PRES recurrence, the potential causative agent was reintroduced to one of the patients who was carefully monitored, as suggested previously.^[10]^

PRES in case 1 was associated with topotecan, an alkaloid derivative of camptothecin commonly used in the treatment of ovarian, cervical, and recurrent lung cancer, which works through the inhibition of the topoisomerase I complex.^[11]^ One of the main toxic effects of topotecan is the depletion of bone marrow, resulting in neutropenia, thrombocytopenia, and anemia.^[12]^ Topotecan can cross the blood–brain barrier; therefore, its use as brain metastases treatment was studied in a phase III trial, but the drug did not show any survival advantage.^[13]^ Topotecan has been associated with aseptic meningitis, seizures, and chronic encephalopathy when administered intrathecally.^[14]^ We are not aware of any previous report describing topotecan as an agent associated with PRES; however, the association of PRES with another inhibitor of topoisomerase I has been reported (irinotecan 180 mg/m^2^).^[15]^

Cases 2 and 3 received agents that are more frequently described in the setting of PRES. Although rare, PRES with paclitaxel (80 to 135 mg/m^2^), an agent that arrests cells in G2 and M phases of the cell cycle,^[16,17]^ and gemcitabine (1000 mg/m^2^), an analogue of cytarabine,^[18]^ has been described, even though these drugs do not cross the blood–brain barrier. Combination with other drugs, such as cisplatin, or cancer brain damage may facilitate the penetration of paclitaxel within the endothelium.^[16–18]^ In case 2, the gemcitabine–paclitaxel regimen was associated with PRES. The underlying mechanism is unclear, but acute kidney injury-induced hypertension might cause and/or facilitate endothelial damage.^[19]^ This patient had transient elevation in LDH levels. LDH is an enzyme frequently used as a marker of tissue damage and its elevation has been proposed as a prognostic factor of PRES.^[20]^

Finally, endothelial alteration induced by the inhibition of vascular endothelial growth factor suggests an important role of bevacizumab as a cause of PRES. The most common reported side effect of bevacizumab is hypertension resulting from nitric oxide release, which disrupts the blood–brain barrier and leads to PRES. A favorable prognosis in patients with bevacizumab-induced PRES has been suggested, and some have proposed the term benign reversible encephalopathy syndrome instead of PRES. ^[21,22]^

In conclusion, PRES is a reversible disorder that can be induced by several chemotherapeutic agents. There is no specific treatment for PRES other than eliminate or treat the precipitating cause. Prognosis is usually favorable; most patients achieved clinical recovery in 2 to 8 days.10% to 20% of patients are reported with persistent neurological sequelae.^[2]^

## Acknowledgments

The authors thank Enago (www.enago.com) for the English language review.

## Author contributions

**Conceptualization:** Bernardo Cacho-Díaz.

**Data curation:** Nydia A Lorenzana-Mendoza, Roberto Corona-Cedillo, Salvador Escobar-Ceballos.

**Methodology:** Bernardo Cacho-Díaz.

**Resources:** Nydia A Lorenzana-Mendoza, Carlos Castillo-Rangel.

**Supervision:** Bernardo Cacho-Díaz, Carlos Castillo-Rangel.

**Validation:** Bernardo Cacho-Díaz, Gervith Reyes-Soto, Jaime G de la Garza-Salazar.

**Visualization:** Roberto Corona-Cedillo.

**Writing – original draft:** Nydia A Lorenzana-Mendoza, Salvador Escobar-Ceballos.

**Writing – review & editing:** Karen Salmerón-Moreno, Gervith Reyes-Soto, Jaime G de la Garza-Salazar.

Bernardo Cacho-Díaz orcid: 0000-0001-9289-5312.

Nydia A. Lorenzana-Mendoza orcid: 0000-0001-5456-6999.
